# Youth organizations, social mobility and health in middle age: evidence from a Scottish 1950s prospective cohort study

**DOI:** 10.1093/eurpub/ckac144

**Published:** 2022-10-26

**Authors:** L Berrie, L Adair, L Williamson, C Dibben

**Affiliations:** School of GeoSciences, University of Edinburgh, Edinburgh, UK; Scottish Centre for Administrative Data Research, University of Edinburgh, Edinburgh, UK; Research Data Scotland, Bayes Centre, Edinburgh, UK; School of GeoSciences, University of Edinburgh, Edinburgh, UK; Scottish Centre for Administrative Data Research, University of Edinburgh, Edinburgh, UK; School of GeoSciences, University of Edinburgh, Edinburgh, UK; Scottish Centre for Administrative Data Research, University of Edinburgh, Edinburgh, UK

## Abstract

**Background:**

Informal educational programmes focused on youth development appear to improve health and well-being at time of involvement. Less is known about long-term effects. We investigate their impact on self-reported general health in mid-life using the Aberdeen Children of the 1950s (ACONF) cohort.

**Methods:**

We use a subset (*n* = 1333) of the ACONF cohort, born 1950–56, in Aberdeen Scotland, who took part in Family and Reading Surveys in 1964 and a follow-up questionnaire in 2001. We explore exposure to youth development focused clubs in childhood on self-reported general health around age 50 mediated by adult socioeconomic position. Logistic regression and mediation analysis were used to report odds ratios and natural direct and indirect effects, respectively, on multiply imputed data.

**Results:**

Being a member of the Scouts/Guides (G&S) was associated with a 53% (95% confidence interval 1.03–2.27) higher odds of ‘excellent’ general health in adulthood compared to children attending ‘other clubs’. Indirect effects of G&S and Boys’/Girls’ Brigade (B&GB) on general health acting via higher socioeconomic position show positive associations; 12% and 6% higher odds of ‘excellent’ general health in adulthood compared to children attending ‘other clubs’, respectively. Comparison of indirect with direct effects suggests 27% of this association is mediated through a higher adult socioeconomic position in adulthood.

**Conclusions:**

These results suggest a beneficial association between attending G&S and B&GB clubs in childhood and adult general health. As these organizations are volunteer-led, this may represent a cost-effective method for improving population health.

## Introduction

Informal educational programmes aimed at helping young people develop capabilities and resilience, particularly those focused on a strengths-based perspective or ‘positive youth development’ (PYD) have been shown to have a beneficial effect on outcomes in youth.^1^ PYD programmes aim to build: social, vocational and cognitive competence; self-confidence; connection to others; respect for societal and cultural rules; and caring and compassion (summarized as the ‘five Cs’ of competence, confidence, character, connection and caring^2^). A youth is ‘thriving’ when they exhibit the ‘Cs’ through time and they are progressing towards ‘idealised adulthood’. Important features of an ideal adult life are ‘marked by integrated and mutually reinforcing contributions to self’, within this is the ability to maintain one’s own health so that one is able to ‘remain an active agent in one’s own development’.^3^ The PYD approach sees all young people as having the ability to develop positive qualities and socially desirable characteristics. This is in contrast to the ‘deficit perspective’ model, which saw adolescents as problems to be managed and positive outcomes focused on the avoidance or reduction of poor choices or behaviours.^4^^,^^5^

Although PYD only emerged as a recognized approach in the 1990s, there is a longer history of structured programmes for children and young people also focused on developing strengths. Organizations such as the Jewish Lads Brigade, Scouts, Guides, Boys’ Brigade (BB) and Girls’ Guildry (GG) developed over the 20th century in the UK with large proportions of children attending one of these organizations. The Guides and Scouts (G&S), BB and GG all had and have detailed programmes and standards governed by regional oversight. Particularly after the reforms of these programmes in the 1960s and 70s, there are clear parallels with the aims of PYD.^6^^,^^7^

Research into youth development programmes has tended to focus on short rather than long-term outcomes.^8^ Research on members of the Scouts have found engagement in the movement to be a predictor of moral and performance character^9^ and prolonged participation in Scouts to be positively associated with active civic engagement in young adulthood.^10^ There is also evidence that PYD might reduce youth substance use and violence.^11^ Previous research in the UK, tested whether G&S attendance was associated with later mental health, controlling for childhood risk factors and social class, and found that G&S had an 18% lower odds [95% confidence interval (95% CI): 8–26% lower odds] of a mood or anxiety disorder at age 50.^12^ A study of attendees of Boy Scouts of America also found evidence of a long-term effect on subjective well-being in adulthood.^13^

Any relationship with informal education in youth and later life health may be mediated by socioeconomic position in adulthood. Socioeconomic status in childhood has been found to have a direct effect on a range of health and well-being measures in adulthood.^14^ Formal education may also ‘fix’ the detrimental effects of low socioeconomic position at birth on self-reported multimorbidity in later life via achieved adulthood socioeconomic position.^15^ It has also been suggested that childhood cognitive ability, socioeconomic position and education act indirectly via adult socioeconomic position to affect psychological health in mid-life.^16^ It is therefore important to explore both the direct and mediated (indirect) effect of childhood conditions on later life health.

In this study, we focus on a long-term outcome, self-reported general health in mid-life and whether this is affected by informal education, specifically, membership of development-oriented clubs in youth and if this occurs through socioeconomic position achieved in adulthood. Specifically we ask (i) is attendance at Guides/Scouts/Cubs/Brownies or BB/Lifeboys/Guildry independently associated with self-reported general health in mid-life, (ii) is this relationship mediated by social position in adulthood and (iii) how does this compare with the impact of extra years spent in education beyond the age of 16?

## Methods

### Cohort and sample

**Figure 1:**
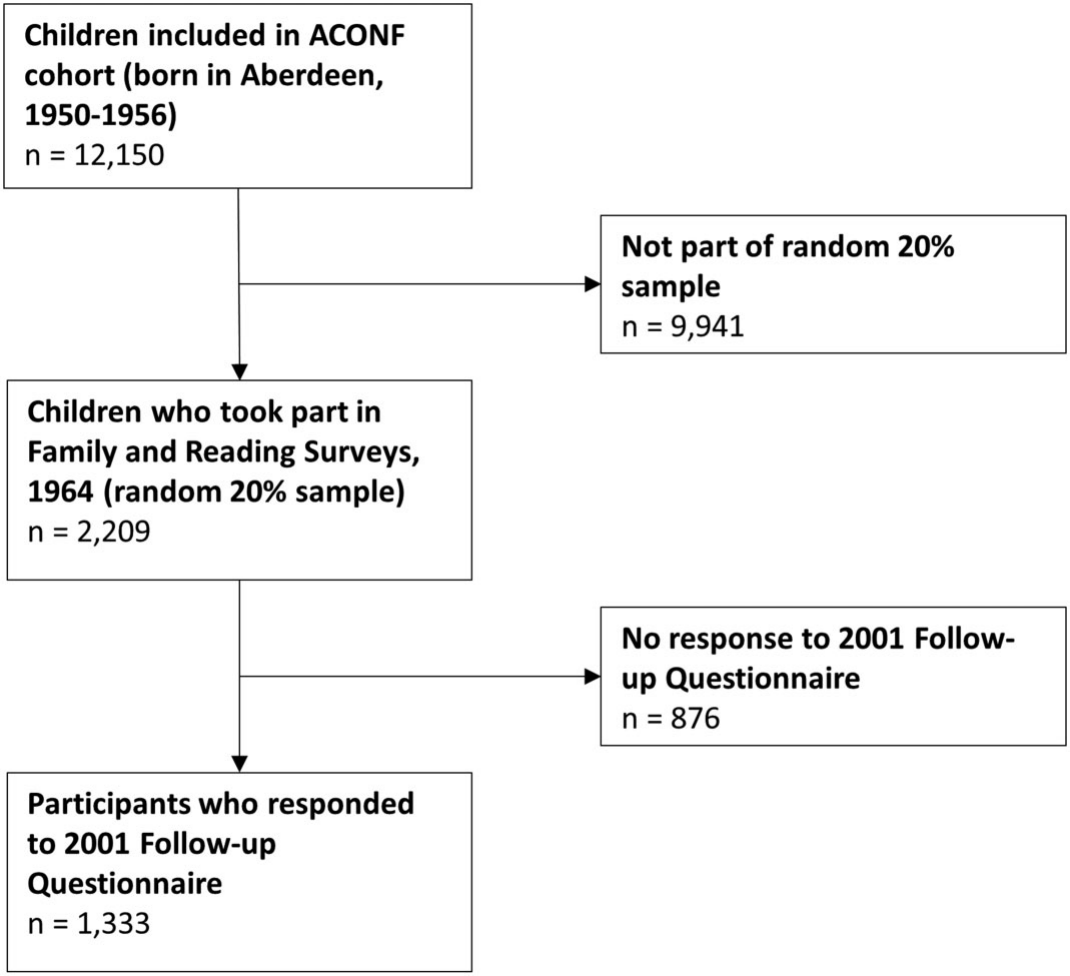
Flow diagram illustrating how our cohort was obtained

The Aberdeen Children of the 1950s (ACONF) study follows a cohort of individuals born in Aberdeen between 1950 and 1956.^17^ Those aged 6–12 years at primary school in Aberdeen were surveyed in December 1962 (*N* = 12 150). Early-life data were obtained from school records, birth certificates and hospital records. A random 20% of the sample were asked about family circumstances, attitudes and behaviour (*N* = 2209) as part of the Family Survey in 1964. In a follow-up, cohort members were traced at NHS Central Registry in 1999. A postal questionnaire was sent in 2001/2002 when participants were aged between 45 and 51. Of the 2209 random sample who completed the 1964 Family Survey, 1333 responded to the 2001 follow-up questionnaire, which makes up our cohort (figure 1).

### Variables

#### Focal relationship

**Figure 2:**
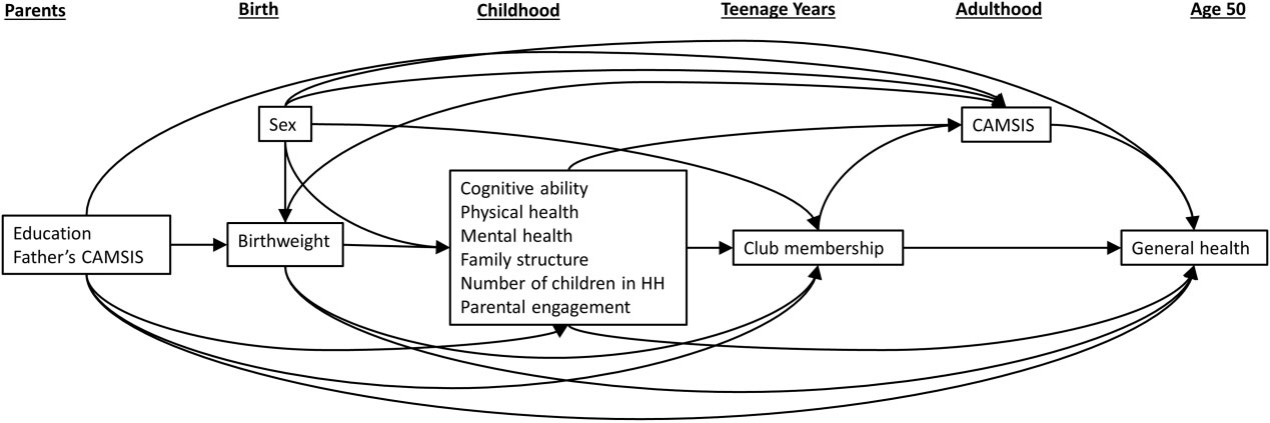
DAG illustrating hypothesized relationships between variables; time runs left to right. Exposure = club membership, outcome = general health, mediator = CAMSIS

This study aimed to estimate the association between participation in youth development focused clubs with general health around age 50 mediated by socioeconomic position in adulthood. Hypothesized relationships between variables are depicted using a directed acyclic graph (DAG) where assumed associations between variables are illustrated using arrows the direction of which represent the assumed direction of association (figure 2).^18^

#### Outcome measure

The outcome measure we assess was general health around age 50 obtained from the 2001 follow-up questionnaire. This was a self-reported measure asking participants: ‘Over the last 12 months would you say that your health on the whole has been…’ with the following choice of response: ‘Excellent’, ‘Good’, ‘Fair’ and ‘Poor’. The responses were grouped to form a dichotomous variable: ‘Excellent’ vs. ‘Good’, ‘Fair’, ‘Poor’. We are particularly interested in ‘best’ health for this relatively young cohort but we also analysed, in sensitivity analysis, the data using a dichotomous variable combining the positive health outcomes (i.e. ‘Good’ and ‘Excellent’) and the negative health outcomes (i.e. ‘Fair’ and ‘Poor’).

#### Exposure measure

ACONF contains data on membership of clubs and youth movements, as well as other club activities in childhood. The questions on club membership were asked of a random 20% followed-up in ‘the Family Survey’ in 1964 when the cohort were aged between 8 and 14 years and was completed by the mothers. We created a variable with the following groups: G&S for those who attended Guides/Scouts/Cubs/Brownies (G&S) even if they attended other clubs; Boys’/Girls’ Brigade (B&GB) for the remainder if they attended BB/Lifeboys/Guildry; ‘other clubs’—anyone who reported that they participated in a youth club; choir; sports but not G&S or B&GB; and No Club if none was indicated.

We also modelled school leaving age as an exposure to allow for comparison between formal and informal education. Specifically, we calculated the difference in general health of those who left formal education at 16 compared to those who carried on beyond 16.

#### Mediator

We consider each participant’s social position on the Cambridge Social Interaction and Stratification (CAMSIS) scale, based on their occupation reported in 2001 in a follow-up questionnaire, as a mediator between club membership and adult general health. CAMSIS is a measure of occupational position based on social interaction patterns between occupations. The underlying theory of CAMSIS suggests that the social distance between occupations, as determined by analysing social interaction patterns, can be used to represent relative social position. The scores range from 1 (least advantaged) to 99 (most advantaged) with a mean of 50 and standard deviation of 15 in the national population.^19^^,^^20^

#### Confounders

There are a number of aspects of a child’s early life that might plausibly influence their chances of attending youth development focused clubs whilst also potentially affecting social position and general health in adulthood. These include socioeconomic situation in childhood, a child’s health, well-being and development and parental support and engagement in development. We set out how we believe these factors might confound the relationships of interest in a DAG (figure 2) and from this identify which variables need to be adjusted for in our models:

Childhood socioeconomic situation

- father’s CAMSIS score (a continuous variable used to measure social position);
- parental further education (a dichotomous variable indicating whether either of the child’s parents attended post-school education).

Health, well-being and development

- child’s birth weight (a dichotomous variable indicating whether the child was born weighing <5.5lbs as a proxy for foetal development);
- child’s Cognitive Ability Score aged 7 (a categorical variable with groupings of: 60–99, 100–109, 110–119, 120–129, 130+) as measured on the Moray House Picture Intelligence Test;
- child physical health (a dichotomous variable indicating whether the child has any physical health problems);
- child mental health (a dichotomous variable indicating whether the child has any mental or emotional health problems).

Parental support and engagement

- family structure (a categorical variable indicating whether the child comes from a two parent, single parent or other family background);
- number of children in the household; and
- parental help with homework (a categorical variable with the following groupings: no help, checks homework and helps with homework as a proxy for parental engagement/a supportive home environment).

We use ‘other clubs’ as the comparison group for our analysis, as we consider those who were participants of ‘other clubs’ to come from backgrounds more like those who participated in youth development clubs, e.g. they come from backgrounds where club participation was considered a worthwhile and valuable pursuit and had families ready to support them in this endeavour and therefore also likely to be supportive more generally. However, we assume that these ‘other clubs’ do not have the active ingredients that groups such as G&S have, which would lead to social mobility and general health in adulthood.

Under the assumptions necessary for conducting mediation analysis, we adjust for confounders affecting the exposure and outcome, the mediator and outcome and the exposure and mediator. We assume that none of the mediator-outcome confounders are affected by the exposure.^21^^,^^22^

#### Missingness

Of the 1333 members of our cohort, 153 (11.5%) were missing at least one variable of interest. We used multiple imputation using chained equations to impute values for the missing data, creating 10 complete datasets. We present the patterns of missingness in Supplementary figure S1 and the total number of missing values for each variable are presented in table 1 alongside the descriptive statistics for all 1333 cohort members. The statistical analyses presented in this article are based on the multiply imputed datasets with results from complete case analyses available (Supplementary tables S1–S10).

**Table 1:** Description of the mediator, outcome and selected confounding variables according to club membership

	Total population *n* = 1333		**Boys’ Brigade/Lifeboys/Guildry** ^a^ ***n* = 303**		**Scouts/Guides/Cubs/Brownies** ^a^ ***n* = 336**		Other clubs *n* = 272		No clubs *n* = 422	
	Mean	Standard deviation	Mean	Standard deviation	Mean	Standard deviation	Mean	Standard deviation	Mean	Standard deviation
Child’s CAMSIS	37.79	19.62	37.92	18.40	46.08	20.10	34.20	19.10	33.34	18.30
Father’s CAMSIS	26.35	17.10	23.77	14.87	32.60	19.23	25.91	17.89	23.31	14.73
	*N*	%	*N*	%	*N*	%	*N*	%	*N*	%
General health										
Excellent	338	29.01	89	29.37	109	32.44	63	23.16	111	26.30
Other	827	70.99	214	70.63	227	67.56	209	76.84	311	73.70
Sex										
Male	625	46.89	194	64.03	114	33.93	119	43.75	198	46.92
Female	708	53.11	109	35.97	222	66.07	153	56.25	224	53.08
Birth weight										
Low	75	5.63	12	3.96	16	4.76	15	5.51	32	7.58
Not low	1254	94.07	290	95.71	319	94.94	256	94.12	389	92.18
Missing	4	0.30	1	0.33	1	0.3	1	0.37	1	0.24
IQ score (age 7)										
60–99	333	24.98	65	21.45	46	13.69	77	28.31	145	34.36
100–109	299	22.43	82	27.06	63	18.75	63	23.16	91	21.56
110–119	283	21.23	73	24.09	72	21.43	58	21.32	80	18.96
120–129	230	17.25	53	17.49	80	23.81	31	11.40	66	15.64
130+	149	11.18	28	9.24	62	18.45	30	11.03	29	6.87
Missing	39	2.93	2	0.67	13	3.87	13	4.78	11	2.61
Parental further education										
None	926	69.47	210	69.31	175	52.08	202	74.26	339	80.33
Further education	401	30.08	91	30.03	158	47.02	69	25.37	83	19.67
Missing	6	0.45	2	0.66	3	0.89	1	0.37	0	0.00
Family structure										
Parents married	1220	91.52	276	91.09	317	94.34	245	90.07	382	90.52
Single parent	73	5.48	18	5.94	14	4.17	17	6.25	24	5.69
Other	26	1.95	8	2.64	3	0.89	6	2.21	9	2.13
Missing	14	1.05	1	0.33	2	0.60	4	1.47	7	1.66
Number of children										
1	153	11.48	34	11.22	46	13.69	28	10.29	45	10.66
2	437	32.78	103	33.99	118	35.12	88	32.35	128	30.33
3	366	27.46	75	24.75	101	30.06	78	28.68	112	26.54
4	204	15.30	48	15.84	52	15.48	41	15.07	63	14.93
5	102	7.65	25	8.25	12	3.57	24	8.82	41	9.72
6+	68	5.10	16	5.28	7	2.08	12	4.41	33	7.82
Missing	3	0.23	2	0.67	0	0.00	1	0.37	0	0.00
Parental help with homework										
No help	307	23.03	70	23.10	59	17.56	84	30.88	94	22.27
Checks	574	43.06	120	39.60	164	48.81	96	35.29	194	45.97
Helps	430	32.26	112	36.96	110	32.74	87	31.99	121	28.67
Missing	22	1.65	1	0.33	3	0.89	5	1.84	13	3.08
Child physical health										
Physical health issue	33	2.48	6	1.98	8	2.38	11	4.04	8	1.90
No physical health issue	1300	97.52	397	98.02	328	97.62	261	95.96	414	98.1
Child mental health										
Mental health issue	64	4.80	13	4.29	10	2.98	14	5.15	27	6.40
No mental health issue	1269	95.20	290	95.71	326	97.02	258	94.85	395	93.60

a Both Boys’ Brigade/Lifeboys/Guildry and Scouts/Guides/Cubs/Brownies groups include children who also attended other groups.

### Statistical analyses

Logistic regression was initially used to model the outcome, participant’s general health score in adulthood and exposure, club membership, whilst adjusting for confounders identified *a priori* with the aid of the DAG (figure 2). We similarly modelled school leaving age as the comparative exposure. Causal mediation analysis under the counterfactual framework was employed to partition the total effect of club membership on adult general health acting through adult socioeconomic position [the natural indirect effect (NIE)] and the effect of club membership acting directly on adult general health [the natural direct effect (NDE)]. We used the imputation method to calculate the NDE and NIE using logistic regression and the logit link function. Non-parametric bootstrapping (1000 repetitions) was used to estimate 95% Confidence Intervals (CIs) of the NDE and NIE. Comparisons were made between the reference group, membership of ‘other clubs’ and each of the G&S, B&GB and ‘None’ group memberships. We report effect estimates and 95% CIs from models of the exposure–mediator and mediator–outcome relationships in the Supplementary materials.

All analyses were conducted using R^23^ version 4.1.0; the mediation analysis using the medflex package^24^ and the multiple imputation using the mice package.^25^

## Results

### Characteristics of the cohort

In total, 336 (114 boys and 222 girls) of our cohort (25.2%) were members of the G&S and 303 (194 boys, 109 girls) were members of the B&GB (22.7%) (table 1). These were mutually exclusive categories; however, 40.7% of those in G&S were in at least one other club as were 38.3% of B&GB. Those who were in G&S came from backgrounds with higher father’s CAMSIS scores, smaller families, had higher cognitive ability, parents who had further education and were more likely to help with homework (table 1).

### Logistic regression model

Regressing club membership on participant’s general health as an adult whilst adjusting for confounders identified by the DAG (figure 2) suggests an increased probability of reporting excellent health around age 50 in those who attended G&S [odds ratio (OR): 1.53; 95% CI: 1.04–2.22] compared to those who attended ‘other clubs’. For B&GB, the odds of reporting excellent health around age 50 compared to those who attended ‘other clubs’ was 1.28 (95% CI: 0.87–1.88). The 95% CIs for the estimated ORs of attending B&GB contained 1 (table 2).

**Table 2:** Estimates of the direct, indirect, total effects expressed as ORs of the association between club membership and ‘excellent’ general health, mediated by adult CAMSIS score conducted on 10 multiply imputed datasets (*N* = 1333)

Club	Direct effect (95% CI)	Indirect effect (95% CI)	Total effect (95% CI)
‘Other clubs’	(Ref)	(Ref)	(Ref)
Boys’ Brigade/Lifeboys/Guildry	1.20	1.06	1.28
	(0.81, 1.79)	(1.00, 1.12)	(0.86, 1.90)
Scouts/Guides/Cubs/Brownies	1.36	1.12	1.53
	(0.92, 2.01)	(1.04, 1.21)	(1.03, 2.27)
No clubs	1.17	1.01	1.19
	(0.80, 1.70)	(0.97, 1.06)	(0.82, 1.72)

*Note*: Adjusted for: father’s CAMSIS score, sex, birth weight, cognitive ability aged 7, child physical health, child mental health, family structure, number of children in the household and parental help with homework. All comparisons are between the club listed and ‘other clubs’. 95% CI, 95% confidence interval.

### Mediation analysis

The results of the mediation analysis conducted on the 10 multiply imputed datasets are shown in table 2. The NDE of club membership on general health (an OR comparing members of each of B&GB and G&S with ‘other clubs’) were 1.20 (95% CI: 0.81–1.79) and 1.36 (95% CI: 0.92–2.01), respectively. The NIE expressed as ORs (in the same order as the NDE) were 1.06 (95% CI: 1.00–1.12) and 1.12 (95% CI: 1.04–1.21), respectively. Using the formula [NIE/(NIE+NDE)],^26^ the proportion of the total effect mediated was estimated as 27% for both B&GB and for G&S.

### Comparator analysis

We analysed the association between years spent in formal education on self-reported general health in our multiply imputed ACONF datasets (Supplementary tables S11 and S12). The OR of reporting excellent health when attending school past 16 years of age compared to leaving school at 16 or younger was 1.23 (95% CI: 0.89–1.7). This suggests that, if either G&S (compared to attending ‘other clubs’) and school leaving age have a causal effect, that the impact on general adult health of attending G&S (compared to attending ‘other clubs’) was greater than the impact of attending school past the age of 16 (compared to leaving school at 16 years of age or younger) and a similar effect to attending B&GB (compared to attending ‘other clubs’).

## Discussion

This study found evidence for a positive association between attending youth development clubs in childhood and youth and adult health. This relationship existed after adjustment for conditions in childhood that affect both later life health and the likelihood of club attendance. This positive association is larger in groups participating in G&S than B&GB. Around a quarter of this association appears to result from the achievement of a higher social position in adulthood. The size of the association on general health for G&S compared to children attending ‘other clubs’ appears quite large compared to other social interventions, such as formal education. The odds of excellent health in adulthood for G&S attenders compared to those attending ‘other clubs’ is about double that of children staying on in school post the age of 16 compared to those leaving at age 16.

Although the UK’s school-based education system aims to produce a fair, meritocratic, distribution of socioeconomic position later in life, there is growing evidence that it does not achieve this goal.^27^ One important factor appears to be the access socioeconomically advantaged young people have to resources that develop non-cognitive skills outside of the formal education system and the protection this offers them to failure within the school system and early life through ‘compensatory advantage’^28^ and then the benefit it offers them in competing in the labour market after leaving education.^29^ Several mechanisms have been proposed as to how youth development programmes could improve social mobility. These include the broadening of social networks to aid social advantage, the acquisition of psychological capital (including resilience, self-efficacy and optimism required for competitive workplace advantage) and the acquisition of social and human capital (leading to stronger job prospects and subsequent social mobility).^30^ In our study, we find the association with later life social position and health remains after controlling for socioeconomic position in childhood, suggesting that rather than being a tool for re-enforcing early-life privilege, youth development groups equally support children from all backgrounds.

We found that some of the association of club membership acts via adult social position. However, a substantial proportion of the measured association seems to have been through other pathways. Based on the findings in the literature on PYD, positive psychological, human and social capital and the long-term benefits of physical activity and the adoption of recreational lifestyles of taking part in clubs are other feasible ways in which later life general health could benefit directly from club membership.^13^ More research is required to understand the components of this relationship.

### Strengths and limitations

This study is based on a cohort recruited at one location—Aberdeen, Scotland—who were born in a short time period (1950–56). Aberdeen in the late 1950s and early 1960s, when the ACONF cohort were participating in clubs, was very different to how it is now; the discovery of a large oil field in the North Sea in the 1970s and subsequent arrival of a new industry brought many changes.^31^ Indeed, this cohort may have been among the first to work in the oil industry, which may affect generalizability of our study to other locations or the present day.

A particular strength of our study is that we have club participation recorded at the time of the activity, rather than retrospectively when reporting of attendance may be affected by recall bias.^32^ However, we do not have information on how long each participant attended the clubs and it has been reported that length of attendance and commitment could be important for outcomes, such as adult civic engagement and social capital.^33^ Research has also reported the largest increases in moral and performance character in highly engaged Scout members (Boy Scouts of America) enrolled in highly engaged packs.^9^

Our study used a self-reported measure of general health in which participants were asked to report on their health over the previous 12 months. This does not differentiate between physical and mental health and simply asks participants to report on their ‘health’, however, our results are in keeping with the other study reporting on Scout–Guide membership and adult mental health.^12^

Recent research has proposed the possible benefits to social mobility of participating in sport, especially when coaches and clubs ‘recognise the pivotal role that they undertake in… generating learning opportunities beyond the sporting domain’.^34^ As it was not possible for us to ascertain the nature of the sports clubs that our cohort participated in, we decided to combine those who only took part in sports with those in ‘other clubs’. As a result, our assumption that ‘other clubs’ are just capturing a supportive home environment, not any active development process, may be wrong. However, if this were the case, it would have biased our results towards a null finding not a false positive effect.

Our analysis was limited to the 20% random sample of the ACONF cohort who took part in the Family Survey (for data regarding club membership) and of those, cohort members who responded to the 2001 follow-up questionnaire (for data regarding socioeconomic position and adult general health). As many participants were missing from this cohort, we were unable to complete multiple imputation for those who did not respond to the questionnaire. Research into the response to the ACONF postal questionnaire found that the traced and non-traced groups were similar in most respects, however, a range of childhood characteristics were associated with the response rate—increasing with cognitive test scores^35^ and advantage in adulthood.^17^ We were able, however, to use missing data techniques for the participants missing covariates. We report the results based on the multiply imputed datasets and include the analysis based solely on the complete cases in the Supplementary material. There is no difference between our conclusions based on either approach.

We have used previous literature and knowledge of the ACONF setting to inform our assumptions of temporal precedence between our variables of interest (as illustrated in our DAG; figure 2). We used the CAMSIS score as a measure of socioeconomic position. Although the CAMSIS score is time-varying, we only have a measure of this at one point in time. In models of how early-life SEP affects adult health, theories have been suggested which highlight the importance of timing, accumulation and change in this relationship.^36^ There may be bias in our analysis due to our inability to account for this, however, our study is an important contribution, which can guide future, more complex life-course models in this area.

In conclusion, the current study examined the association between childhood club attendance with self-reported general health in mid-life and its pathway via adult socioeconomic position using data from the ACONF cohort. We found evidence that participation in clubs in childhood that can be considered to have a PYD focus is associated with self-reported adult general health with this relationship being partly mediated by adult social position. This adds to previous research in this area, which found a positive relationship between Scout and Guide participation and adult mental health^12^ and suggests that further research into the long-term effects of other organized activities in youth may be useful. Because many of the organizations that deliver these youth programmes are charities supported by volunteers, they may represent a very cost-effective method of delivering population health.

## Supplementary data

Supplementary data are available at *EURPUB* online.

## Supplementary Material

ckac144_Supplementary_Data

## Data Availability

Data can be shared with accredited researchers upon application to the ACONF Steering Committee with appropriate approvals in place. Informal education programmes focused on youth development have been found to be related to better health outcomes in youth. This study finds evidence of a positive association between engagement with informal educational organizations in childhood and later life health, an association that appears larger than the benefit of staying on in school post age 16. As many of the organizations that deliver these youth programmes are charities supported by volunteers, they may represent a very cost-effective method of improving population health.
