# Supplementary material for: Youth organizations, social mobility and health in middle age: evidence from a Scottish 1950s prospective cohort study
Source: Eur J Public Health. 2022 Oct 26;33(1):6–12. doi: 10.1093/eurpub/ckac144 (PMC9898007; doi:10.1093/eurpub/ckac144)
Supplement: ckac144_Supplementary_Data [file ckac144_supplementary_data.zip › ckac144_Supplementary_Data/ejph-2022-04-om-0227-File004.docx]

## Supplementary Material for Youth organisations, social mobility, and health in middle age. Evidence from a Scottish 1950s prospective cohort study

### Additional descriptive data

Supplementary Figure Captions

Figure S1: Missingness map of variables included in modelling

Figure S2: Distribution of (A) father’s and (B) child’s CAMSIS scores at the 2001 follow-up questionnaire by club membership.

### Models on original dataset – complete case analysis

#### Exposure-mediator model

Table S1: Effect estimates and 95% confidence intervals of the association between club membership and adult CAMSIS score conducted on the original dataset (N = 1179). Linear regression adjusted for: sex, birthweight, Cognitive Ability aged 7, child physical health, child mental health, family structure, number of children in the household, and parental help with homework. All comparisons are between the Club listed and ‘Other’ club.

| Club | EFFECT Estimate | 95% Confidence Interval |
| --- | --- | --- |
| Boys’ Brigade/lifeboys/guildry | 3.39 | 0.59 to 6.19 |
| Scouts/Guides/CUBs/brownies | 6.44 | 3.67 to 9.21 |
| none | 0.89 | -1.70 to 3.49 |

#### Mediator-outcome model

Table S2: Effect estimates and 95% confidence intervals expressed as odds ratios of the association between adult CAMSIS score and general health conducted on the original dataset (N = 1179). Logistic regression adjusted for: club membership, sex, birthweight, Cognitive Ability aged 7, child physical health, child mental health, family structure, number of children in the household, and parental help with homework.

| exposure | EFFECT Estimate (Odds ratio) | 95% Confidence Interval |
| --- | --- | --- |
| camsis score | 1.016 | (1.01, 1.02) |

#### Mediation analysis

Table S3: Estimates of the direct, indirect, total effects expressed as odds ratios of the association between club membership and general health, mediated by adult CAMSIS score conducted on the original dataset (N = 1179). Adjusted for: sex, birthweight, Cognitive Ability aged 7, child physical health, child mental health, family structure, number of children in the household, and parental help with homework. All comparisons are between the Club listed and ‘Other’ club.

| Club | direct effect  *(95% CI)* | Indirect effect  *(95% CI)* | total effect  *(95% CI)* |
| --- | --- | --- | --- |
| Boys’ Brigade/lifeboys/guildry | 1.40  *(0.91, 2.13)* | 1.05  *(1.00, 1.12)* | 1.48  *(0.96, 2.24)* |
| Scouts/Guides/CUBs/brownies | 1.60  *(1.04, 2.38)* | 1.11  *(1.03, 1.19)* | 1.77  *(1.16, 2.64)* |
| none | 1.33  *(0.90, 1.94)* | 1.02  *(0.98, 1.07)* | 1.36  *(0.92, 1.98)* |

### Models with alternative general health dichotomy (i.e. Excellent and Good vs. Fair and Poor) – complete case analysis

#### Logistic regression model

Table S4: Effect estimates and 95% confidence intervals of the association between club membership and adult general health with the dichotomy Excellent and Good vs Fair and Poor, conducted on conducted on the original dataset (N = 1179). Logistic regression adjusted for: sex, birthweight, Cognitive Ability aged 7, child physical health, child mental health, family structure, number of children in the household, and parental help with homework. All comparisons are between the Club listed and ‘Other’ club.

| Club | EFFECT Estimate | 95% Confidence Interval |
| --- | --- | --- |
| Boys’ Brigade/lifeboys/guildry | 1.53 | 0.99 to 2.38 |
| Scouts/Guides/CUBs/brownies | 1.98 | 1.24 to 3.20 |
| none | 1.74 | 1.15 to 2.63 |

#### Exposure-mediator model

Table S5: Effect estimates and 95% confidence intervals of the association between club membership and adult CAMSIS score conducted on the original dataset (N = 1179) and adult general health dichotomy Excellent and Good vs Fair and Poor. Linear regression adjusted for: sex, birthweight, Cognitive Ability aged 7, child physical health, child mental health, family structure, number of children in the household, and parental help with homework. All comparisons are between the Club listed and ‘Other’ club.

| Club | EFFECT Estimate | 95% Confidence Interval |
| --- | --- | --- |
| Boys’ Brigade/lifeboys/guildry | 3.54 | 0.55 to 6.54 |
| Scouts/Guides/CUBs/brownies | 6.96 | 3.94 to 9.97 |
| none | 1.43 | -1.39 to 4.24 |

#### Mediator-outcome model

Table S6: Effect estimates and 95% confidence intervals expressed as odds ratios of the association between adult CAMSIS score and general health with the dichotomy Excellent and Good vs Fair and Poor, conducted on the original dataset (N = 1179). Logistic regression adjusted for: club membership, sex, birthweight, Cognitive Ability aged 7, child physical health, child mental health, family structure, number of children in the household, and parental help with homework.

| exposure | EFFECT Estimate (Odds ratio) | 95% Confidence Interval |
| --- | --- | --- |
| camsis score | 1.02 | (1.01, 1.03) |

#### Models on multiply imputed datasets

Table S7: Effect estimates and 95% confidence intervals of the association between club membership and adult general health with the dichotomy Excellent and Good vs Fair and Poor, conducted on conducted on 10 multiply imputed datasets (N = 1333). Logistic regression adjusted for: sex, birthweight, Cognitive Ability aged 7, child physical health, child mental health, family structure, number of children in the household, and parental help with homework. All comparisons are between the Club listed and ‘Other’ club.

| Club | EFFECT Estimate | 95% Confidence Interval |
| --- | --- | --- |
| Boys’ Brigade/lifeboys/guildry | 1.47 | 0.97 to 2.22 |
| Scouts/Guides/CUBs/brownies | 1.93 | 1.24 to 3.00 |
| none | 1.54 | 1.06 to 2.25 |

#### Exposure-mediator model

Table S8: Effect estimates and 95% confidence intervals of the association between club membership and adult CAMSIS score conducted on ten multiply imputed datasets (N = 1333) and adult general health dichotomy Excellent and Good vs Fair and Poor. Linear regression adjusted for: sex, birthweight, Cognitive Ability aged 7, child physical health, child mental health, family structure, number of children in the household, and parental help with homework. All comparisons are between the Club listed and ‘Other’ club.

| Club | EFFECT Estimate | 95% Confidence Interval |
| --- | --- | --- |
| Boys’ Brigade/lifeboys/guildry | 3.39 | 0.59 to 6.19 |
| Scouts/Guides/CUBs/brownies | 6.44 | 3.67 to 9.21 |
| none | 0.89 | -1.70 to 3.49 |

#### Mediator-outcome model

Table S9: Effect estimates and 95% confidence intervals expressed as odds ratios of the association between adult CAMSIS score and general health with the dichotomy Excellent and Good vs Fair and Poor, conducted on ten multiply imputed datasets (N = 1333). Logistic regression adjusted for: club membership, sex, birthweight, Cognitive Ability aged 7, child physical health, child mental health, family structure, number of children in the household, and parental help with homework.

| exposure | EFFECT Estimate (Odds ratio) | 95% Confidence Interval |
| --- | --- | --- |
| camsis score | 1.02 | 1.01 to 1.03 |

#### Mediation analysis

Table S10: Estimates of the direct, indirect, total effects expressed as odds ratios of the association between club membership and general health with the dichotomy Excellent and Good vs Fair and Poor, mediated by adult CAMSIS score conducted on 10 multiply imputed datasets (N = 1333). Adjusted for: sex, birthweight, Cognitive Ability aged 7, child physical health, child mental health, family structure, number of children in the household, and parental help with homework. All comparisons are between the Club listed and ‘Other’ club. CI = Confidence Interval.

| Club | direct effect  *(95% CI)* | Indirect effect  *(95% CI)* | total effect  *(95% CI)* |
| --- | --- | --- | --- |
| Boys’ Brigade/lifeboys/guildry | 1.36  *(0.89, 2.10)* | 1.07  *(1.00, 1.15)* | 1.47  *(0.95, 2.28)* |
| Scouts/Guides/CUBs/brownies | 1.69  *(1.07, 2.67)* | 1.14  *(1.05, 1.25)* | 1.93  *(1.21, 3.07)* |
| none | 1.50  *(1.03, 2.21)* | 1.02  *(0.96, 1.09)* | 1.54  *(1.04, 2.28)* |

### Education as exposure models

#### Original health dichotomy (Excellent vs. Good, Fair, Poor) – complete case analysis

Table S11: Estimate of the effect, expressed as an odds ratio, of the association of school leaving age and general health on the original dataset (N = 1179). Adjusted for: father’s CAMSIS score, sex, birthweight, Cognitive Ability aged 7, child physical health, child mental health, family structure, number of children in the household, and parental help with homework. Comparison between leaving school after the age of 16 or before.

| School leaving age | Odds of reporting excellent health  *(95% CI)* |
| --- | --- |
| leaving school at 16 years of age or younger | 1 |
| attending school past 16 years of age | 1.26  *(0.90, 1.76)* |

#### Original health dichotomy (Excellent vs. Good, Fair, Poor) – analysis of MI datasets

Table S12: Estimate of the effect, expressed as an odds ratio, of the association of school leaving age and general health on the multiply imputed datasets (N = 1333). Adjusted for: father’s CAMSIS score, sex, birthweight, Cognitive Ability aged 7, child physical health, child mental health, family structure, number of children in the household, and parental help with homework. Comparison between leaving school after the age of 16 or before.

| School leaving age | Odds of reporting excellent health  *(95% CI)* |
| --- | --- |
| leaving school at 16 years of age or younger | 1 |
| attending school past 16 years of age | 1.23  *(0.89, 1.70)* |
