# Supplementary figures and images for: Youth organizations, social mobility and health in middle age: evidence from a Scottish 1950s prospective cohort study
Source: Eur J Public Health. 2022 Oct 26;33(1):6–12. doi: 10.1093/eurpub/ckac144 (PMC9898007; doi:10.1093/eurpub/ckac144)

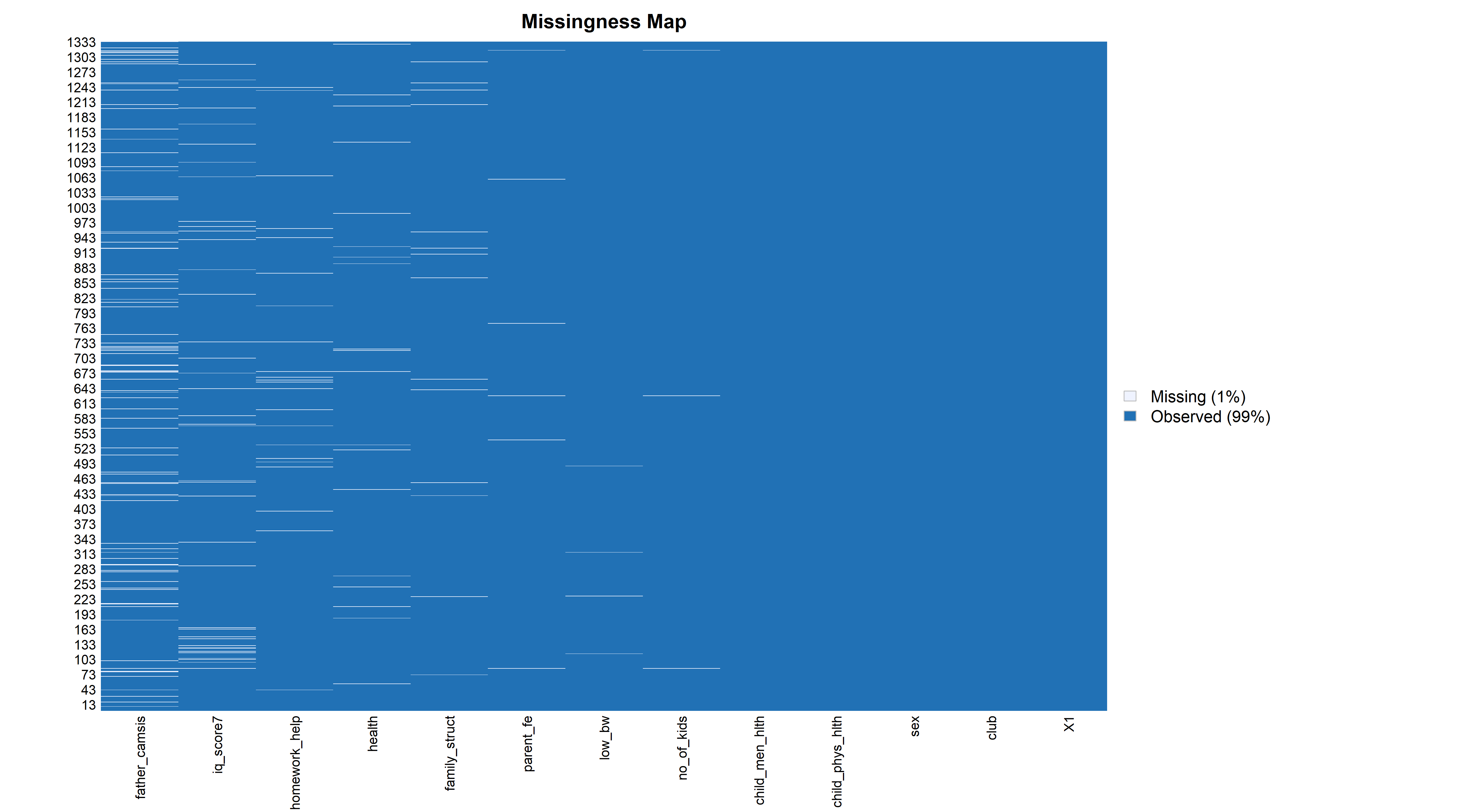

Supplement: ckac144_Supplementary_Data [file ckac144_supplementary_data.zip › ckac144_Supplementary_Data/ejph-2022-04-om-0227-File005.tif]
